# New Insights into the Androgen-Targeted Therapies and Epigenetic Therapies in Prostate Cancer

**DOI:** 10.1155/2011/918707

**Published:** 2011-10-12

**Authors:** Abhijit M. Godbole, Vincent C. O. Njar

**Affiliations:** ^1^Department of Pharmaceutical Sciences, Jefferson School of Pharmacy, Thomas Jefferson University, 130 South 9th Street, Edison Building, Suite 1510F, Philadelphia, PA 19107, USA; ^2^Department of Pharmacology and Experimental Therapeutics, University of Maryland and School of Medicine, 685 West Baltimore Street, Baltimore, MD 21201-1559, USA; ^3^Kimmel Cancer Center, Thomas Jefferson University, Philadelphia, PA 19107, USA

## Abstract

Prostate cancer is the most common cancer in men in the United States, and it is the second leading cause of cancer-related death in American men. The androgen receptor (AR), a receptor of nuclear family and a transcription factor, is the most important target in this disease. While most efforts in the clinic are currently directed at lowering levels of androgens that activate AR, resistance to androgen deprivation eventually develops. Most prostate cancer deaths are attributable to this castration-resistant form of prostate cancer (CRPC). Recent work has shed light on the importance of epigenetic events including facilitation of AR signaling by histone-modifying enzymes, posttranslational modifications of AR such as sumoylation. Herein, we provide an overview of the structure of human AR and its key structural domains that can be used as targets to develop novel antiandrogens. We also summarize recent findings about the antiandrogens and the epigenetic factors that modulate the action of AR.

## 1. Introduction

Prostate cancer (PC) is the second most prevalent cause of death in men in the USA and Europe. The dependence of PC on androgens has been recognized for more than 7 decades. Medical and surgical androgen deprivation therapy (ADT) has been a standard palliative therapy for metastatic PC. However, an estimated 217,730 new cases and 32,050 PC-related deaths in the USA alone in 2010 despite ADT [1] make the need for finding new targets and novel therapies an absolute priority. 

Androgen, the male steroid hormone, is responsible for male sexual differentiation and development, as well as the maintenance and support of sexual tissues in the adult. Moreover, androgens are important for the development and progression of age-associated pathologies in men, including benign prostatic hyperplasia and prostate cancer (PC). Androgen action is exerted through the androgen receptor (AR), a 110-kDa member of the steroid receptor family of transcription factors [2]. The physiological ligands for the AR are testosterone and dihydrotestosterone (DHT). The later has at least 10-fold stronger binding affinity.

The most commonly used therapies in metastatic prostate cancer involve androgen deprivation through medical (LHRH agonists), surgical castration, or disruption of androgen binding to AR [3]. Such treatments are temporarily effective, but, over time, most prostate cancers evolve into a castration-resistant state [4, 5]. Resistance mechanisms include AR, gene mutation or amplification, ligand independent activation of AR and persistent intraprostatic androgens [6–8]. Importantly, even in castration resistant prostate cancer (CRPC), AR still plays an essential role in cancer progression [6]. Recent work indicates that epigenetic enzymes are important coactivators of AR and may represent targets to influence AR stability and activity, thus providing new therapeutic opportunities to overcome mechanisms of resistance.

Histone-deacetylating and DNA-methylating enzymes, act to modify certain histone and nonhistone proteins such as the chaperone protein HSP90, which leads to enhanced protein stability of client proteins including AR [9–11]. Due to the central role of AR in all phases of prostate cancer, modulating AR protein stability or AR cofactor activity represents an effective strategy to overcome most of the mechanisms of resistance and may have therapeutic implications in this disease.

This paper discusses the structure of androgen receptor, current antiandrogen therapies, the emerging therapies that target the AR, epigenetic modulation of AR, and therapies targeting epigenetic modulation.

## 2. Androgen Receptor (AR)

AR is a nuclear hormone receptor, which is activated by binding of androgen ligands. Upon androgen binding, AR dissociates from the cytoplasmic chaperone protein HSP90, self-dimerizes and translocates to the nucleus. AR then binds to consensus sequences in the genome called AREs (androgen response elements) to activate transcription of its target genes, which is essential for prostate development and maintenance [12].

**Structure of AR**

**Figure 1:**
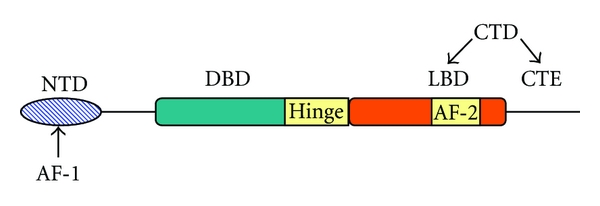
Schematic representation of the structure of human androgen receptor NTD: N-terminal domain, DBD: DNA-binding domain, LBD: ligand-binding domain, CTE: C-terminal extension, CTD: C-terminal domain, AF-1: activation function-1, AF-2: activation function-2.

The AR shares an overall modular organization which has an N-terminal domain (NTD) containing the activation function (AF)-1, a central DNA binding domain (DBD), a short hinge region, and a COOH terminal domain (CTD), which contains both the AR ligand-binding domain (LBD) and AF-2 coactivator binding surface (Figure 1) [13]. The three-dimensional structures of peptides representing the LBD and AF-2 folds of the AR have been determined by X-ray crystallography. The three-dimensional structure of a peptide representing the AR DBD has also been determined [14, 15]. The AR NTD, on the contrary, is unstructured in solution, and thus it has been difficult to predict its structure accurately. Nevertheless, several critical functional domains have been described and characterized within the AR NTD. Posttranslational modifications of the AR, including phosphorylation, acetylation, ubiquitylation, and sumoylation, add additional layers of regulation and are likely to influence the structure and function of these domains [16] (Figure 1).

### 2.1. AR C-Terminal Domain (CTD)

The role of the AR CTD is of particular importance for PC, because the current androgen ablation therapies target this domain of the AR. This prevents both AR nuclear translocation and the exposure of AF-2. Antiandrogens such as bicalutamide bind the LBD, block the activity of AF-2 [12], and cause AR to recruit corepressor molecules such as nuclear receptor corepressor (NCoR) to the promoters of AR-regulated genes [17, 18]. This inhibits AR activity and thus halts the growth and survival of androgen-dependent PC cells. Binding of ligand to the AR LBD causes a conformational change in the AR CTD, which induces formation of the AF-2 coactivator binding surface. The AF-2 surface serves as a docking site for LxxLL motifs present in transcriptional coactivators and corepressors. Unlike the LBD of other nuclear receptors, the AR LBD displays very weak ligand-dependent transcriptional activity unless stimulated by p160 coactivators such as steroid receptor coactivator (SRC)-1 or SRC-2 [19, 20]. This is in absolute contrast to the potent inherent transcriptional activity of an isolated AR NTD fragment [20–22]. 

The transcriptional activity of most steroid hormone receptors is predominantly through the activation function AF-2 region in the LBD. However, in the AR, it is the AF-1 region in the NTD that contributes most to the transcriptional activity [23]. AR LBD functions independently of the NTD and can still bind ligand even if the AF-1 region is deleted or mutated; however, AF-1 region in the NTD is an absolute requirement for the transcriptional activity to take place.

### 2.2. AR N-Terminal Domain (NTD)

The AR NTD is highly flexible and displays intrinsic disorder in solution, which has hampered elucidation of its three-dimensional structure [13, 24]. In other words, it represents a rigid secondary structure which is either exposed or concealed depending on various factors such as androgen levels, cell type, posttranslational modifications, and presence or absence of transcriptional modulators. The AR NTD accounts for more than 60% of the AR protein. Therefore, understanding the dynamic nature of the AR NTD is critically important. It functions as a potent transcriptional activator independent of the CTD. The AF-1 domain serves as a binding site for the transcriptional repressors- N-CoR (nuclear corepressor) and SMRT (silencing mediator of retinoic acid and thyroid hormone receptor). Sumoylation (SUMO-small ubiquitin-like modifier) is a type of posttranslational modification. Sumoylation by SUMO-1 protein requires two discrete lysine residues—K386 and K520. These modifications inhibit AR activity through a mechanism that may involve SMRT's interaction with the AR NTD [25, 26]. To sum up, the AR NTD plays a multifunctional and dynamic role in regulating AR activity and is a very important target.

### 2.3. AR-DBD

The AR DBD/hinge region plays important roles in mediating AR nuclear localization, receptor dimerization, and DNA binding. All the hormone receptor DBDs are highly conserved. It consists of two zinc-fingers and a loosely structured carboxy-terminal extension (CTE) [27]. It is responsible for dimerization of AR and tight binding of AR dimer to the DNA.

Ligand binding induces a conformational change in AR inducing phosphorylation [28], nuclear translocation [29], and dimerization. As described above, AR dimmer then binds to androgen response elements (AREs) located in the regulatory regions of target genes [30] and actively recruits essential cofactors and assembles the transcriptional machinery required to regulate the expression of androgen-regulated genes [31, 32]. A critical component of this signaling axis is the ability of the receptor to undergo dimerization.

### 2.4. AR Dimerization

Dimerization mediated through the AR-DBD is an absolute requirement for AR signaling. By assembling on DNA targets, the AR homodimer specifically binds DNA to regulate the expression of AR target genes. Whereas the N/C (NTD/CTD) interaction provides a potential mechanism for regulation of AR activity, further studies are necessary to determine the precise role and contribution of each model of AR N/C interaction to AR signaling. Based on evidence that AR-LBD dimerization can occur, it is likely that this interaction also contributes to AR signaling. Centenera et al. showed that interaction of multiple AR domains is required for optimal AR-mediated signaling [33].

## 3. Therapies Targeting Androgen Signaling

**Figure 2:**
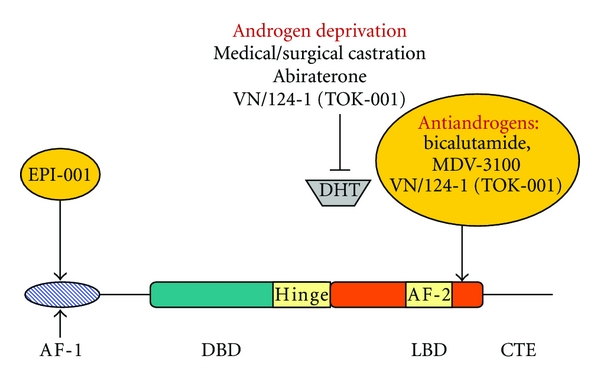
Therapeutic approaches to block the AR. EPI-001 interacts with the AR NTD to block AR transcriptional activity through this domain. Inhibitors of the LBD include androgen ablation and antiandrogens, DHT, and dihydrotestosterone.

Currently, all conventional therapy has been focused on androgen-dependent activation of the AR through its C-terminal LBD. A schematic of their mechanism of action is shown in (Figure 2). 

Below is a summary of the currently used therapies targeting androgen signaling.

### 3.1. Androgen Deprivation Therapy (ADT)—Castration

Several studies have attempted to surgically or pharmacologically target androgenic stimulation. The goal of these interventions is to slow disease progression, and to treat the disease. *Surgical castration* completely eliminates testosterone production by the testes, whereas administration of an *LHRH agonist* (medical castration) generates castrate levels of serum testosterone (<20 or <50 ng/dL, resp.) by having a negative hormonal feedback on the hypothalamus [34]. There was no statistically significant difference in disease-free or overall survival for metastatic patients treated with either of the these testosterone-lowering treatments [35]. Castration was associated with a number of adverse effects like hot flashes, loss of libido, and decreased quality of life.

### 3.2. CYP17 Inhibitors

**Figure 3:**
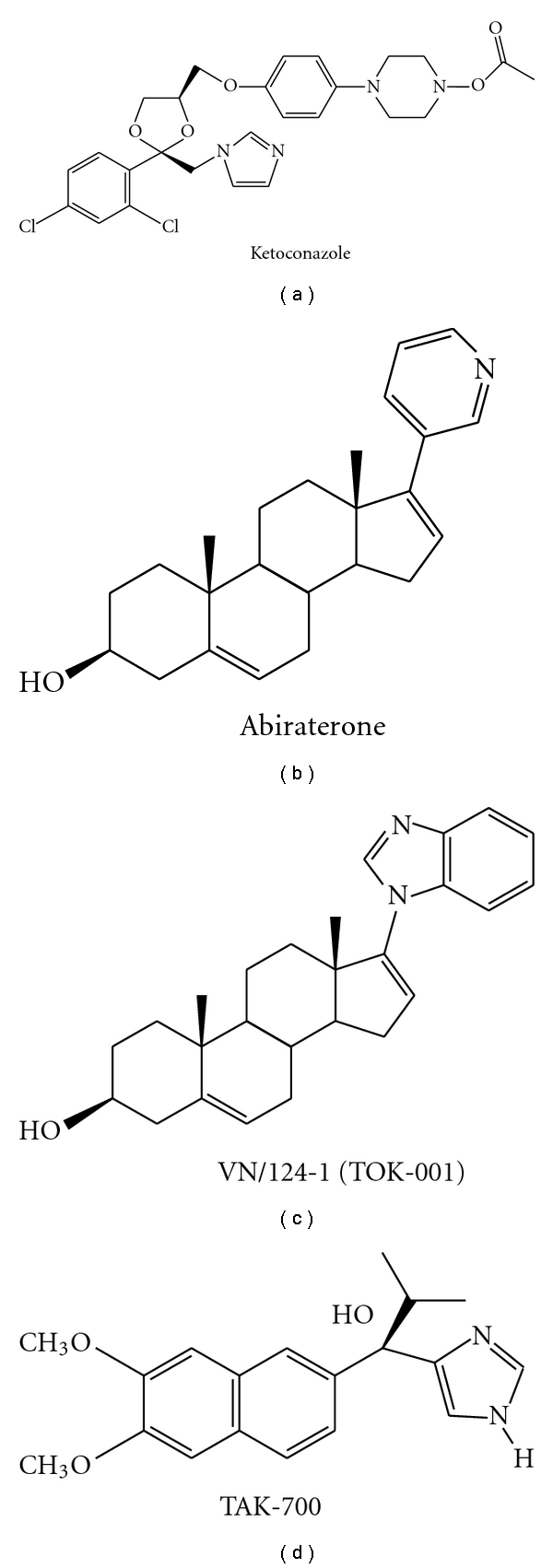
Structures of Inhibitors of CYP17.

Blocking the *in situ* production of androgens by inhibition of CYP 17 enzyme is a critical key in the treatment of patients with advanced and/or metastatic prostate cancer. The following section describes the currently used CYP17 inhibitors and relevant data about them in clinical trials. The structures of ketoconazole, abiraterone acetate and VN/124-1 (TOK-001) are presented in Figure 3. 

#### 3.2.1. Ketoconazole

Ketoconazole is a broad spectrum antifungal agent that has been extensively used off-label as second-line hormonal therapy for prostate cancer. Ketoconazole inhibits 11-*β* hydroxylation, cholesterol side chain cleavage to pregnenolone and CYP17 [36]. Two single-center trials on the use of HDK in CRPC found PSA declines >50% in 55% (11/20) [37] and 63% (30/48) of patients [38]. A larger phase III study of HDK therapy in 260 patients with post-ADT metastatic PC on antiandrogen withdrawal (AAWD) demonstrated a PSA decline >50% in 27% of patients treated with HDK plus AAWD. Overall survival was not different between the treatment groups; however, those patients with a >50% PSA decline had a median survival of 41 months compared to 13 months for those without a PSA decline. Time to PSA progression in PSA responders was 5.9 *versus* 8.6 months in AAWD alone and AAWD + HDK groups, respectively [39]. Androstenedione, dehydroepiandrosterone (DHEA), and dehydroepiandrosterone sulfate (DHEAS) levels decreased with HDK therapy. However, there was no change in testosterone level from baseline in either treatment groups.

#### 3.2.2. Abiraterone Acetate

Abiraterone, a highly selective irreversible CYP17 inhibitor, was developed as a mechanism-based steroidal inhibitor of CYP17 following observations that nonsteroidal 3-pyridyl esters had improved selectivity for inhibition [40]. Abiraterone has been shown to reduce serum testosterone levels to below a detection threshold of 1 ng/dL [41]. Promising results from clinical trials of abiraterone acetate in CRPC patients have recently been reported. In a phase I trial of abiraterone acetate treatment of both ketoconazole-pretreated and ketoconazole-naïve CRPC patients [42], PSA declines of ≥50% were seen in 18 (55%) of 33 patients, including nine (47%) of 19 patients with prior ketoconazole therapy and nine (64%) of 14 patients without prior ketoconazole therapy. Significantly, the antitumor activity was nearly equivalent in both populations. The activity observed in castrate, ketoconazole-naïve patients confirms that abiraterone acetate is an active agent, whereas the activity in ketoconazole pre-treated patients implies that a more selective and potent inhibitor of CYP17 may be an improvement beyond ketoconazole, or an additional sequential therapeutic option.The most common adverse events in patients treated with abiraterone acetate were fatigue, hypertension, headache, nausea, and diarrhea. In addition to chemotherapy-naïve patients, a multicenter phase II study evaluated the efficacy of abiraterone in patients with docetaxel-treated CRPC [43]. All patients were treated with 1000 mg/d. Forty-seven patients were enrolled, and treatment resulted in observed PSA declines ≥50% in 51% (24/47) of patients at least once. Partial responses (by RECIST criteria) were reported in 27% (8/30) patients with measurable disease. Decreases in circulating tumor cell (CTC) counts were also observed [43].

Two phase III clinical trials of abiraterone acetate are now in progress. The first of these trials is designed to evaluate abiraterone + prednisone against a placebo + prednisone in patients with progressive CRPC after docetaxel chemotherapy. This trial has an estimated study completion date of June 2011 [44]. The second study will evaluate abiraterone + prednisone against a placebo + prednisone in CRPC patients prior to chemotherapy. The estimated study completion date is in 2014. Both trials list prior ketoconazole treatment in their exclusion criteria. Abiraterone was recently approved by US Food and Drug Administration (FDA) in April 2011.

#### 3.2.3. VN/124-1 (TOK-001)

VN/124-1 was rationally designed as an inhibitor of androgen biosynthesis via inhibition of CYP17. Utilizing intact CYP17 expressing *Escherichia coli*, VN/124-1 was shown to be a potent inhibitor of the enzyme with an IC_50_ value of 300 nM compared to abiraterone which had an IC_50_ value of 800 nM. The high efficacy of VN/124-1 in several prostate cancer models is believed to arise from its ability to downregulate the AR as well as competitively block androgen binding. In competitive binding studies against the synthetic androgen [^3^H] R1881, VN/124-1 was equipotent to bicalutamide in LNCaP cells. Transcriptional activation assays showed VN/124-1 to be a pure AR antagonist of the wild-type AR and the T877A mutation found in LNCaP cells [45, 46]. VN/124-1 inhibited the growth of CRPCs, which had increased AR and were no longer sensitive to bicalutamide [47]. 

VN/124-1 (0.13 mmol/kg twice daily) caused a 93.8% reduction (*P* = 0.00065) in the mean final LAPC-4 xenograft volume compared with controls. In another antitumor efficacy study, treatment of VN/124-1 (0.13 mmol twice daily) was very effective in preventing the formation of LAPC4 tumors. VN/124-1 (0.13 mmol/kg twice daily) and VN/124-1 (0.13 mmol/kg twice daily) + castration induced regression of LAPC4 tumor xenografts by 26.55 and 60.67%, respectively [46]. This impressive preclinical data led to further clinical development of VN/124-1 by Tokai Pharmaceutical Cambridge, Mass. Tokai Pharmaceuticals initiated ARMOR1 (Androgen Receptor Modulation Optimized for Response 1) phase 1/2 trials in castrate-resistant prostate cancer patients on November 5, 2009 [48]. The results of this clinical trial are awaited. The study is expected to be completed by July 2012.

### 3.3. AR Antagonists

**Figure 4:**
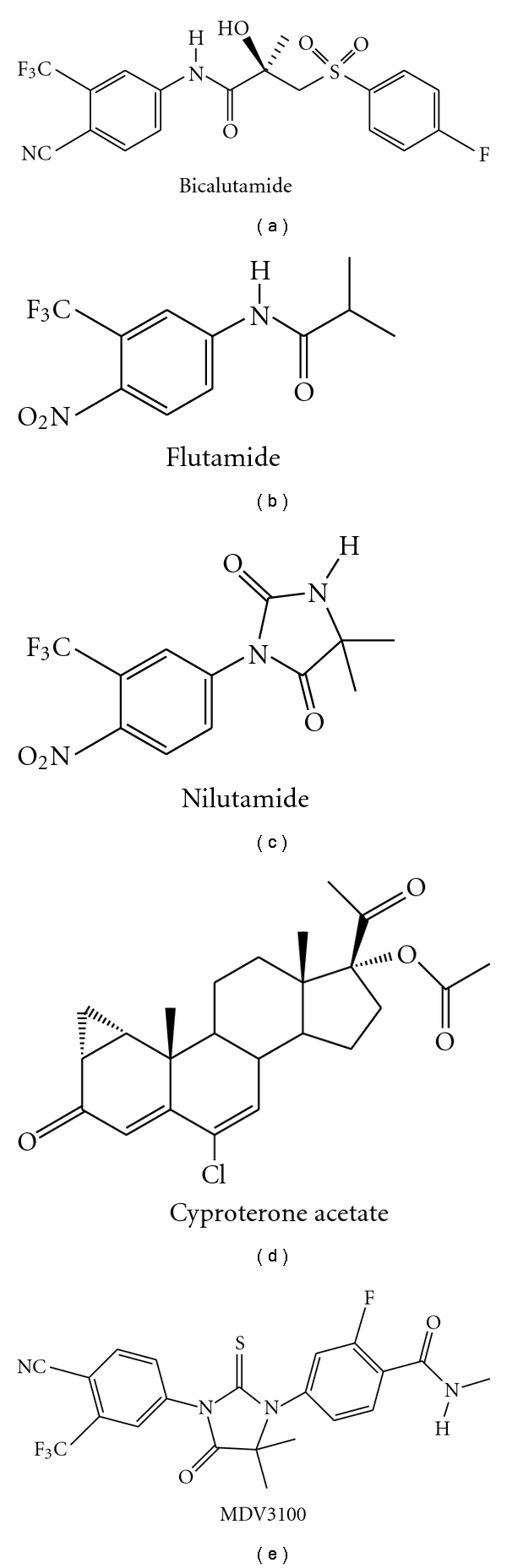
Structures of currently used antiandrogens and clinical candidate MDV3100.

There is ample evidence in the literature that prostate cancer growth can be inhibited by blocking the AR. AR antagonists compete with dihydrotestosterone (DHT) for binding to the AR and thus block AR signaling. Despite the significant reduction in circulating testosterone, castration does not affect adrenal androgen production. Therefore, antiandrogens were introduced to directly prevent the binding of testosterone to the AR. Antiandrogens competitively inhibit ligand binding to the AR and may also prevent ligand-independent AR activation through various pathways, such as inhibiting the recruitment of coactivators or activating corepressors [49]. Antiandrogens are classified as steroidal or nonsteroidal based on their respective chemical structures [50]. The major antiandrogens in clinical use worldwide are the nonsteroidal bicalutamide, flutamide and nilutamide and the steroidal cyproterone acetate (CPA) (Figure 4). Bicalutamide is the most extensively studied nonsteroidal antiandrogen [51]. Lowered percentages of hot flashes as compared with castration have been reported with bicalutamide, flutamide and CPA treatment. Patients treated with bicalutamide have reported better preservation of sexual interest compared with LHRH agonist alone [52]. It is also important to note that a meta-analysis of randomized trials comparing CPA and ADT with ADT alone showed a survival decrease in the CPA group [53]. Overall, the nonsteroidal antiandrogens appear to be better tolerated than castration [54]. Agents targeting AR that are in clinical trials are summarized in Table 1. As monotherapy with an AR antagonist is not yet a standard treatment for patients with advanced or metastatic prostate cancer, it has been combined with medical (or surgical) castration, initially in studies conducted in the late 1980s and early 1990s (complete androgen blockade). These clinical trials showed that the combination of surgical or medical castration plus the administration of an AR antagonist resulted in only a limited improvement in disease-specific and overall survival in patients with advanced and/or metastasized prostate cancer compared to those who receive castration only [55].

Following the evidence that AR expression is increased in CRPC, the diarylthiohydantoin *MDV3100* (Figure 4) was developed as a second-generation antiandrogen capable of sustained AR antagonism under conditions of AR over-expression. In preclinical evaluation, MDV3100 was shown to bind to the AR with a five- to eightfold higher affinity than bicalutamide [18, 56]. In a phase I/II study in CRPC, antitumor activity of MDV3100 was assessed by time on treatment, PSA, soft tissue and osseous disease and circulating tumor cells (CTC). Doses of up to 600 mg/day were investigated. Out of 114 patients treated with 30–360 mg/day and followed for over 12 weeks, 65 were chemotherapy-naïve and 49 were post chemotherapy. At 12 weeks, reduced PSA levels were seen in both groups, with a 57% (37/65) decline in the naïve group and 45% (22/49) in the postchemotherapy patients [18, 57]. No progression was noted in 74% (35/47) of patients with evaluable soft-tissue legions and 62% (50/81) of patients with bone lesions. Dose-limiting toxicity was observed at 600 mg/day. Fatigue was noted at 360 and 480 mg/day. Hence, the dose was reduced. At concentrations of 60, 150, and 240 mg/day, MDV3100 was well tolerated and no serious adverse events related to the drug were reported. Of the 73 patients, 63 had available CTC counts. A total of 85% of those with favorable pretreatment CTC counts maintained favorable posttreatment CTC counts, and 58% of patients treated at 240 mg/day converted from unfavorable to favorable, post-treatment. Bone scans revealed stable disease in 29% (6/21) patients with osseous disease on 240 mg/day. A half-life of 1 week was established, and the current reported data suggest a dose-response trend. Ultimately, 240 mg/day was selected for the phase III trials, and the results are much anticipated.

Although castration and antiandrogens are very effective strategies, it is well known that PC eventually acquires resistance. The possible mechanisms of the resistance include amplification or overexpression of AR; gain-of-function mutations allowing AR to be activated by steroids or antiandrogens, ligand-independent activation by growth factors, cytokines, or kinases; intracrine signaling by increased intratumoral androgens; overexpression of AR coactivators; and/or expression of constitutively active splice variants of AR that lacks LBD may be expressed solely or in mixed populations with full-length receptor to form a heterodimer [58–60]. Although, various spliced variants of AR is reported, Sawyer's group showed that the expression of the spliced variants of the AR are controlled by full-length AR [61]. Clearly, more studies are required to unravel the impact of spliced variants in the development and progression of CRPC.

**Figure 5:**
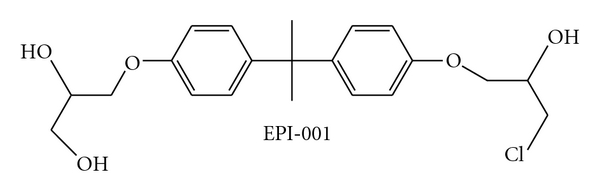
Structure of EPI-001.

For PC that has progressed to a CRPC phenotype, novel strategies of AR inhibition could be based on knowledge regarding the mechanisms of AR activation in this phase of the disease. Several studies have implicated the AR NTD as a key mediator of ligand-independent AR activity in PC cells [62–65]. Quayle et al. showed that a decoy molecule representing the AR NTD inhibited tumor incidence, growth, and hormonal progression in an LNCaP xenograft model of PC [65]. Moreover, intratumor injection of lentivirus expressing the AR NTD decoy fragment inhibited the growth of established LNCaP xenografts. Although preclinical studies about the decoy NTD are impressive, future improvements will rely on more precise modes of inhibition, for example, targeting specific NTD transcriptional activation domains with combinations of smaller peptide decoys or drug-like small molecules. These types of approaches would be greatly facilitated by structural knowledge of the entire AR protein. Decoy AR_1-558_ inhibits full-length AR and blocks both androgen-dependent and CRPC tumor growth, most likely by a mechanism of mopping up essential proteins required for transcriptional activity [65]. Development of shorter decoy peptides (~100 amino acids in length) to the AR NTD that retain specificity for AR and still have antitumor activity has been difficult due to multiple factors, including peptide lability and the possible requirement of multiple, nonlinear regions of the AR NTD necessary for protein-protein interactions. Consistent with inhibiting AR activity, a small molecule inhibitor of AR NTD, EPI-001 (Figure 5) blocks AR-dependent proliferation in human prostate cancer cells that express AR and has no effect on the proliferation of cells that do not express functional AR or do not rely on the AR for growth and survival [66]. 

Intravenous injection of EPI-001 significantly reduced the weight of benign prostates from noncastrated mature mice compared with control-treated animals [66]. EPI-001 also blocked the growth of prostate cancer xenografts in the presence of androgen (noncastrated mature male mice) and, most importantly, caused tumor regression of CRPC. EPI-001 had no effect on PC3 human prostate cancer xenografts that are insensitive to androgen and do not express functional AR indicating that its action is specific for AR proficient cells [66]. 

The aforementioned antiandrogens can have synergistic or additive effects if combined with other antiandrogens or with transcriptional modulators.

## 4. Epigenetic Regulators of AR-Mediated Signaling

To regulate transcription, the receptors bind to specific hormone response elements of target genes and exhibit crosstalk with other transcription factors through protein-protein interactions. Several coregulatory proteins recognized by different functional domains of the AR (the N-terminal transactivation region, the central DNA-binding domain (DBD), and the C-terminal ligand-binding domain) mediate transactivation and transrepression functions of AR and other nuclear receptors [67]. The mechanisms by which steroid receptors compartmentalize in the nuclei and find their specific binding motifs, hormone response elements, from a vast number of base pairs of chromosomal DNA have remained elusive.

Recent work indicates that epigenetic enzymes are important coactivators of AR and may represent targets to affect AR function or stability, thus providing new therapeutic opportunities to overcome mechanisms of resistance and to target AR with nonhormonal therapies. Epigenetics is defined as the study of changes produced in gene expression caused by mechanisms other than changes in the underlying DNA sequence [68]. Among the different types of epigenetic changes, the most important are DNA methylation and histone modifications, both of which have been shown to be important for cancer progression [68]. A histone octamer, composed of two copies of histone H2A, H2B, H3, and H4, is wrapped by approximately 146 base pairs of DNA to form the core particle of a nucleosome, the fundamental structural unit of eukaryotic chromatin [69]. The N-terminal tails of histones extend from the nucleosome core, providing sites for posttranslational modifications such as acetylation, methylation, ubiquitination, and phosphorylation. Such modifications affect chromatin structure and gene transcription [70]. Histone lysine acetylation generally activates gene transcription, whereas lysine methylation can have different effects depending on the position and status of methylation. Methylation of Lys4, Lys36, or Lys79 on histone H3 usually results in transcriptional activation, whereas methylation of Lys9 or Lys27 on histone H3 or Lys20 on histone H4 is usually linked to transcriptional inhibition. On a general note, methylation inhibits gene transcription [68]. The homologues of histone-modifying enzymes, such as HDAC6, may act to deacetylate nonhistone substrates such as the chaperone protein HSP90, which leads to enhanced protein stability of client proteins including AR [9–11, 71]. The balance between repressive and active histone modifications (also known as the histone code) ultimately determines whether a gene will be actively transcribed or repressed.

### 4.1. Histone Deacetylases (HDAC)

HDACs catalyze the deacetylation of the acetylated lysine residues of histones and nonhistone proteins and are involved in various fundamental life phenomena, such as gene expression and cell cycle progression. To date, eighteen HDAC family members (HDAC1—11 and SIRT1—7) have been identified. They are grouped into four classes based on function and homology to their yeast counterparts: class I includes HDAC1, 2, 3, and 8; class II includes HDAC4, 5, 6, 7, 9, and 10; class III includes Sirt1-7 class IV includes HDAC11 [72]. The functions of the HDAC isoforms are not yet fully understood. Some of the HDAC isoforms have been suggested to be associated with various disease states, including cardiac diseases and cancer [73]. 

Some HDACs deacetylate nonhistone substrates. More than 50 nonhistone HDAC substrates have been identified thus far, including key transcription factors such as p53, E2F, alpha-tubulin, and the chaperone protein HSP90 [73]. To sum up, HDACs regulate gene expression by modifying histone and nonhistone proteins. HDACs are upregulated in many cancers. Halkidou et al. showed that HDAC1 is overexpressed in prostate cancer [74]. Another type of HDAC- HDAC6 is an important protein for AR activity. It is described below.

#### 4.1.1. HDAC6

Histone deacetylase 6 (HDAC6) is a cytoplasmic enzyme that regulates many important biological processes, including cell migration, immune synapse formation, and the degradation of misfolded proteins. HDAC6 deacetylates tubulin, Hsp90, and cortactin and forms complexes with other partner proteins. It is the only HDAC that possesses two functional deacetylase domains and a zinc finger (ZnF) motif [75, 76]. In vivo, the enzymatic activity of HDAC6 is exerted on tubulin, heat shock protein 90 (Hsp90), and cortactin substrates; however, in vitro, HDAC6 is also able to deacetylate histones [75–77]. The Zn^2+^ chelator trichostatin A (TSA) reversibly inhibits HDAC6 deacetylase activity [77, 78]. 

AR requires peculiar cellular machinery to achieve the appropriate conformation for binding ligand [79]. Heat shock protein 90 (Hsp90) plays a central role in the formation of a multichaperone complex essential for stabilizing steroid receptors in a conformation receptive to ligand [80, 81]. Hsp90 regulates the AR protein half-life by forming conformation-dependent higher-order chaperone complexes. Hsp90 inhibition prevents the ligand-dependent nuclear translocation of AR, suggesting a role for Hsp90 in the nuclear import of AR [82]. 

 HDAC6 reversibly deacetylates Hsp90, modulating Hsp90 regulation of nuclear receptors, including AR and glucocorticoid receptor (GR) [10, 83, 84]. Inactivation of HDAC6 results in the accumulation of acetylated Hsp90, which no longer forms a stable complex with GR, leading to defective GR ligand binding, nuclear translocation, and transactivation [10]. These findings strongly implicate HDAC6-mediated acetylation/deacetylation of Hsp90 as a potential mechanism regulating steroid hormone signaling. AR forms chaperone complexes with Hsp90 [85]. Recently, it was reported that HDAC6 is required for stabilization of AR protein [86], Ai et al. demonstrated that inactivation of HDAC6 inhibited AR nuclear localization and subsequent transactivation in PC and mouse embryonic fibroblast (MEF) cells [87]. Reexpressing HDAC6 or a deacetylation-mimic Hsp90 mutant alleviated the inhibition. Furthermore, HDAC6 knockdown also inhibited the establishment of C4-2 xenograft tumors in castrated, but not testis-intact, nude mice. These findings together provide evidence for an important role for HDAC6 in AR hypersensitivity and nuclear localization in castration-resistant PC cells, and thus HDAC6 is a very important target which can be exploited in CRPC treatment.

#### 4.1.2. HDAC Inhibitors

**Figure 6:**
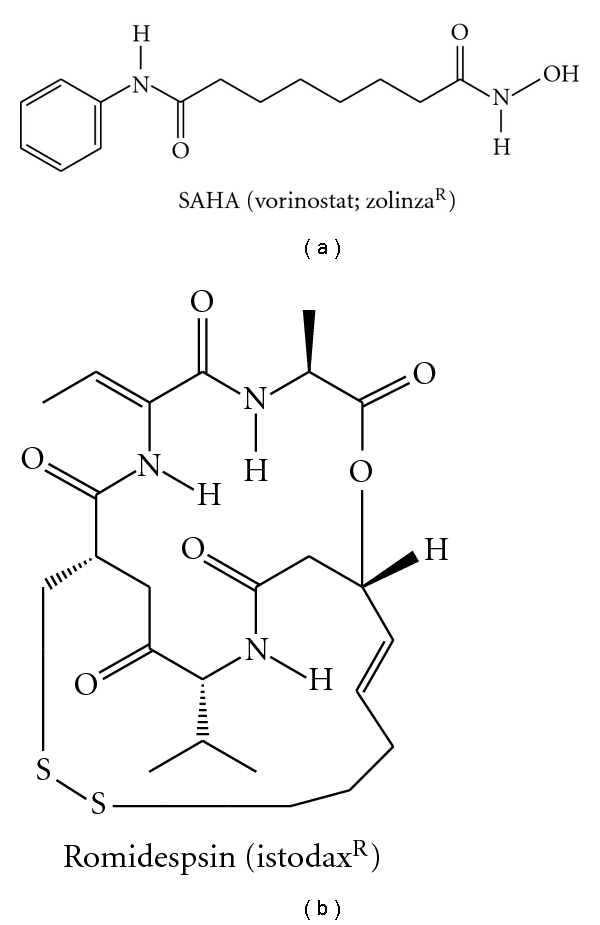
FDA-approved HDAC inhibitors.

To date, SAHA and romidepsin are the only FDA-approved HDAC inhibitors (Figure 6) [88, 89]. 

On a general note, HDAC inhibitors cause histone hyperacetylation, activating many genes, which leads to growth inhibition, differentiation, or apoptosis [73]. Clinical outcome with HDAC inhibitors to fight prostate cancer has yielded disappointing results. However, HDAC inhibitor therapy in prostate cancer still holds great promise due to recent developments. A recent report showed that class I HDACs are essential coactivators of AR (Table 2) [90]. Two widely used HDAC inhibitors, SAHA and LBH589, block transcriptional activation of many AR targets, such as TMPRSS2-ERG [90]. This effect was recapitulated by siRNA to HDAC1, and, to a lesser extent, by siRNA to HDAC3 [90]. Further, Welsbie et al. also showed that these HDAC inhibitors do not block AR recruitment to its targets, rather, they suppress AR target gene activation by blocking the recruitment of AR coactivators and RNA polymerase II [90]. These findings highlight the need for more specific and less toxic HDAC inhibitors in the treatment of PC. 

Some of the HDAC inhibitors serve the dual functions of disrupting AR signaling and reducing AR protein levels in the cell. Multiple reports have shown that HDAC inhibitors suppress AR expression [11, 90–92]. Gibbs et al. reported that sulforaphane—an important constituent in cruciferous vegetarian diet, enhances HSP90 acetylation through HDAC6 inactivation, which leads to disruption of AR binding to HSP90, eventual AR degradation, and reduced expression of AR target genes [93]. Unlike other compounds with HDAC inhibitory function, sulforaphane treatment led to reduced AR binding to its target gene AREs [93]. To sum up, HDAC inhibitors, including compounds such as sulforaphane with effects on HDAC6, inhibit prostate cancer cell growth, which is at least partially explained by effects on AR signaling. Isoform-selective HDAC inhibitors are of great interest as candidate therapeutic agents with few side effects.

Kang et al. [95] showed that transcriptional activation by AR is accompanied by a cascade of distinct covalent histone modifications. Korkmaz et al. [96] studied the role of histone acetylation on AR function. Using three independent HDAC inhibitors: depsipeptide (FR901228), sodium butyrate (NaB), and TSA, they found that inhibition of HDAC activity caused significantly increase in the transcription ability of AR in the LNCaP cell. They found dose-dependent effects of NaB and depsipeptide on AR activity: low doses caused increase in levels of PSA mRNA, whereas high doses of NaB completely inhibited PSA expression. This implies that HDAC inhibitors repress both AR expression and AR-dependent expression of PSA in a dose-dependent manner [96, 97]. Another mechanism of AR suppression by HDAC inhibitors was shown by Welsbie et al. [90]. HDAC inhibitors, vorinostat (SAHA), and LBH589 block AR activity through suppression of the coactivator/RNA polymerase II complex assembly after binding of AR to the promoters of target genes. Rokhlin et al. [91] found that TSA sharply reduced AR gene expression after 24 hours treatment, with partial recovery after 48 hours and returned to normal levels after 72 hours later.

### 4.2. SUMO-1 (Small Ubiquitin-Like Modifier-1)

The SUMO-1 modification (sumoylation) pathway resembles that of ubiquitin conjugation, but the enzymes involved in the two processes are distinct [98]. SUMO-1 (also known as sentrin, GMP1, PIC1, and Ubl1, or in yeast as Smt3) is activated for conjugation by E1 enzymes and subsequently transferred to the E2-conjugating enzyme Ubc9 [99]. Sumoylation is reversible [100]. The sumoylation appears to play multiple roles, including (i) protein targeting, (ii) protein stabilization, and (iii) transcriptional activation. Poukka et al. showed that AR is covalently modified by SUMO-1. They identified that sumoylation sites are present in the N-terminal domain of AR, and they further showed that the SUMO-1 modification in certain contexts indeed inhibits the activity of AR [26].

### 4.3. N-CoR and SMRT

N-CoR and SMRT are well characterized corepressors. Given the evidence that SMRT and NCoR form complexes with HDAC3 [76, 101, 102], these corepressors could have an additive effect on the inhibition of transcription and histone acetylation. However, despite the roles of SMRT and NCoR in regulating the transcriptional activity of several nuclear receptors, the significance of SMRT and/or NCoR on AR transcriptional activity is less clear. Both SMRT and NCoR proteins interact with AR. The AR specific domain binds to the PSA promoter or to various AREs of AR target genes [97, 103]. Trtková et al. analyzed the binding of AR-SMRT complex to PSA promoter and/or binding AR alone to PSA promoter using various NaB concentrations. The ChIP analysis coupled with qPCR of LNCaP and C4-2 cells demonstrated that NaB promoted the formation of the AR-SMRT complex [104]. In summary, N-CoR and SMRT also provide additional epigenetic targets which need to be explored.

### 4.4. Histone Methylation and Demethylases

Histone methylation was originally thought to be a stable, irreversible mark as only histone methyltransferases had been identified with no known data about demethylases. However, the discovery of the first histone demethylase LSD1 (lysine-specific demethylase 1) confirmed that histone methylation is reversible [105, 106]. The status of histone lysine methylation has been shown to be important for AR signaling, and several histone demethylase proteins are upregulated in prostate cancer. These include LSD1 and the Jumonji class of proteins [107–109]. While the transcriptional targets of these proteins are largely unknown, these proteins complex with AR and facilitate its activation of downstream signaling pathways. While LSD1 may demethylate the active dimethyl lysine 4 (2MK4) mark on histone H3, which leads to reduced gene expression of AR-regulated genes, several reports show that LSD1 binds to AREs, the site where AR binds to the DNA and facilitates demethylation of the repressive mono-(1MK9) and dimethyl lysine 9 (2MK9) marks on histone H3 upon recruitment of ligand-bound AR, which results in transcriptional derepression [107, 108]. Given the importance of these enzymes in the activation of AR target genes, inhibition of these enzymes may be a rational, nonhormonal strategy to disrupt AR signaling. MAOIs (monoamine oxidase inhibitors) and polyamine analogues are some of the currently known compounds that inhibit LSD1 activity [107]. These agents need to be further evaluated for their potential to treat prostate cancer.

## 5. Conclusions

Androgen deprivation has been the major therapy for prostate cancer for 7 decades. After a few months of ADT, PC progresses despite castrate serum levels of testosterone. We now have a greater understanding of the mechanisms of sustained AR signaling in these CRPCs that have progressed despite androgen deprivation. Cofactors such as HDACs, SUMO-1, N-CoR, and SMRT provide additional level of control of AR signaling. Upregulation of cofactors such as HDACs that facilitate AR target gene activation is an important event in the development of CRPC. Whether targeting histone deacetylases and other AR coactivators or AR protein stability will be safe and efficacious remains unknown, but their preclinical data holds a great promise. Most of the currently developed therapies targeting AR signaling aim at the LBD of AR. However, with the knowledge that AR NTD can function independent of AR LBD, newer therapies such as EPI-001 and decoy AR NTD are being developed to target AR NTD. All of these antiandrogens and HDAC inhibitors may show synergistic/additive activities when used in combination. There is a great interest to combine these agents to effectively treat CRPC.

## Figures and Tables

**Table 1:** Agents targeting AR in clinical development for CRPC.

Drug	Mechanism of action	Patient characteristics	Phase of development	Clinical trial registration number
MDV-3100	AR antagonist	Chemotherapy-treated	Phase III	NCT00974311
		Chemotherapy-naïve	Phase III	NCT01212991
ARN-509	AR antagonist	ND	Phase I-II	NCT01171898
AZD3514	AR antagonist	ND	Phase I-II	NCT01162395
Abiraterone acetate	CYP 17 inhibitor	Chemotherapy-treated	Phase III	NCT00638690
		Chemotherapy-naïve	Phase III	NCT00887198
Orteronel (TAK-700)	CYP 17 inhibitor	Chemotherapy-treated	Phase III	NCT01193257
VN/124-1 (TOK-001)	AR downregulating agent, CYP 17 inhibitor, and AR antagonist	ND	Phase I-II	NCT00959959

ND: not defined.

**Table 2:** List of HDACs that play a role in AR signaling.

Enzymes	Substrates	Modulation of AR signaling	References
HDAC1	Histones, p53, Smad7, Stat3, and so forth	Facilitates AR-dependent transcription; mechanism not fully clarified	[90, 94]
HDAC3	Histones, Smad7, Stat3, SRY, NF*κ*B, and so forth	Facilitates AR-dependent transcription; mechanism not fully clarified	[90, 94]
HDAC6	Alpha-tubulin, HSP90	Deacetylates HSP90 which enhances chaperoning of AR protein	[9, 10, 93]
